# Association Between Discharge Medications and Oncologic Post‐Embolization‐Syndrome‐Related Outcomes

**DOI:** 10.1002/cam4.71418

**Published:** 2025-12-03

**Authors:** Hanzhou Li, John Moon, Nathan Sim, Michal Horný, Nicholas Lima, Menelaos Konstantinidis, Deepak Iyer, Sonia Benenati, Judy Gichoya, Janice Newsome, Zachary Bercu

**Affiliations:** ^1^ Department of Radiology and Imaging Sciences Emory School of Medicine Atlanta Georgia USA; ^2^ Department of Radiology Brigham and Women's Hospital, Harvard Medical School Boston Massachusetts USA; ^3^ Department of Health Promotion and Policy, School of Public Health and Health Sciences University of Massachusetts Amherst Amherst Massachusetts USA; ^4^ Geisel School of Medicine at Dartmouth Hanover New Hampshire USA; ^5^ University of Toronto, Institute of Health Policy, Management and Evaluation Toronto Ontario Canada; ^6^ Department of Diagnostic, Molecular and Interventional Radiology Icahn School of Medicine at Mount Sinai New York New York USA

**Keywords:** chemoembolization, hepatocellular carcinoma, opioids, post‐embolization syndrome, radioembolization, readmission

## Abstract

**Background:**

Post‐embolization syndrome after transarterial chemoembolization (TACE) and Yttrium‐90 radioembolization (TARE) causes significant morbidity. Understanding whether discharge prescriptions influence short‐term outcomes may guide standardized pain‐management strategies.

**Methods:**

A retrospective cohort study of 3191 patients (3988 procedures) with hepatocellular carcinoma from the Merative MarketScan Databases (2009–2022) was performed. The composite outcome was 7‐day drug escalation or hospital readmission. Bivariate logistic regression identified candidate variables (*p* < 0.10); multivariable logistic regression with patient‐clustered robust standard errors estimated adjusted odds ratios (aORs), adjusting for age, sex, and Charlson Comorbidity Index (CCI).

**Results:**

Compared to patients discharged without opioids post‐chemoembolization, those prescribed opioids at discharge had 83% lower odds of experiencing drug escalation or readmission (odds ratio [aOR] = 0.17, *p* < 0.001), and those undergoing radioembolization had 59% lower odds (aOR = 0.41, *p* < 0.001). Being prescribed antiemetics or steroids was also associated with lower odds of escalation/readmission events, with percentages varying by procedure type.

**Conclusions:**

Prescribing opioids, along with antiemetics and steroids, at discharge may reduce the likelihood of post‐procedural events, such as drug escalation and readmission, in patients undergoing trans‐arterial chemoembolization and radioembolization for hepatocellular carcinoma. These findings highlight the importance of a comprehensive pain management strategy in interventional oncology and warrant consideration in clinical practice guidelines.

## 1 Introduction

Hepatocellular carcinoma (HCC) is the 5^th^ most frequent cancer and the 2^nd^ most common cause of cancer‐related mortality globally [1]. Advancements in the treatment of HCC through minimally invasive and locoregional therapies, such as trans‐arterial chemoembolization (TACE) and yttrium‐90 trans‐arterial radioembolization (TARE), have elevated interventional oncology (IO) as the fourth pillar of oncologic treatment [2] with its integration into early stages of liver cancer treatment paradigms in the National Comprehensive Cancer Network and Barcelona Clinic Liver Cancer guidelines [3, 4].

However, pain continues to be a prevalent, distressing, and undertreated consequence of cancer and its therapeutic interventions [5, 6]. The primary pharmacologic basis for contemporary pain management paradigms stems from the analgesic ladder proposed by the World Health Organization (WHO) in 1986 [7]. Despite these pharmacologic strategies, as much as 32% of cancer patients in developed countries, such as the US, are estimated to be undertreated, and this may be secondary to restrictive prescription practices that evolved in response to the US opioid epidemic [8]. In the context of cancer care, such practices should not exacerbate existing inadequate management of cancer‐related pain. With respect to TACE and TARE, these interventions are used to bridge patients to transplant, improve quality of life, and increase overall survival [9]. However, a frequent consequence of these intra‐arterial treatments includes post‐embolization syndrome (PES), which is characterized by right upper quadrant abdominal pain and fever with possible nausea and vomiting [10]. Although variable in severity, PES‐related abdominal pain has reported incidences of 43% for TACE [11] and 19.8%–20.2% for TARE [12]. Post‐embolization pain may result in significant discomfort for patients, which can lead to hospital readmissions, increased length of stay, and even increased risk of death [10, 13]. PES has been better studied in uterine fibroid embolization (UFE) [14], and efforts to mitigate UFE‐related PES have been published; however, there remains no clear consensus on best practices to treat pain, optimize patient discharge, and decrease pain‐related admissions [14]. The present study investigates the association between prescription of opioid pain medications at the time of discharge and PES‐related outcomes.

## 2 Methods

### 2.1 Study Design

We conducted a retrospective cohort study using the Merative MarketScan Commercial and Medicare Supplemental Databases, which are comprised of deidentified records of between 20 and 50 million patients annually under commercial health plans or covered by employer‐sponsored Medicare supplemental plans. This study was approved by the institutional review board. This investigation adhered to the Strengthening the Reporting of Observational Studies in Epidemiology (STROBE) guidelines.

### 2.2 Setting

We extracted data on the use of inpatient and outpatient services and prescription drugs among patients who underwent IO procedures from January 2009 through June 2022.

### 2.3 Participants

**FIGURE 1:**
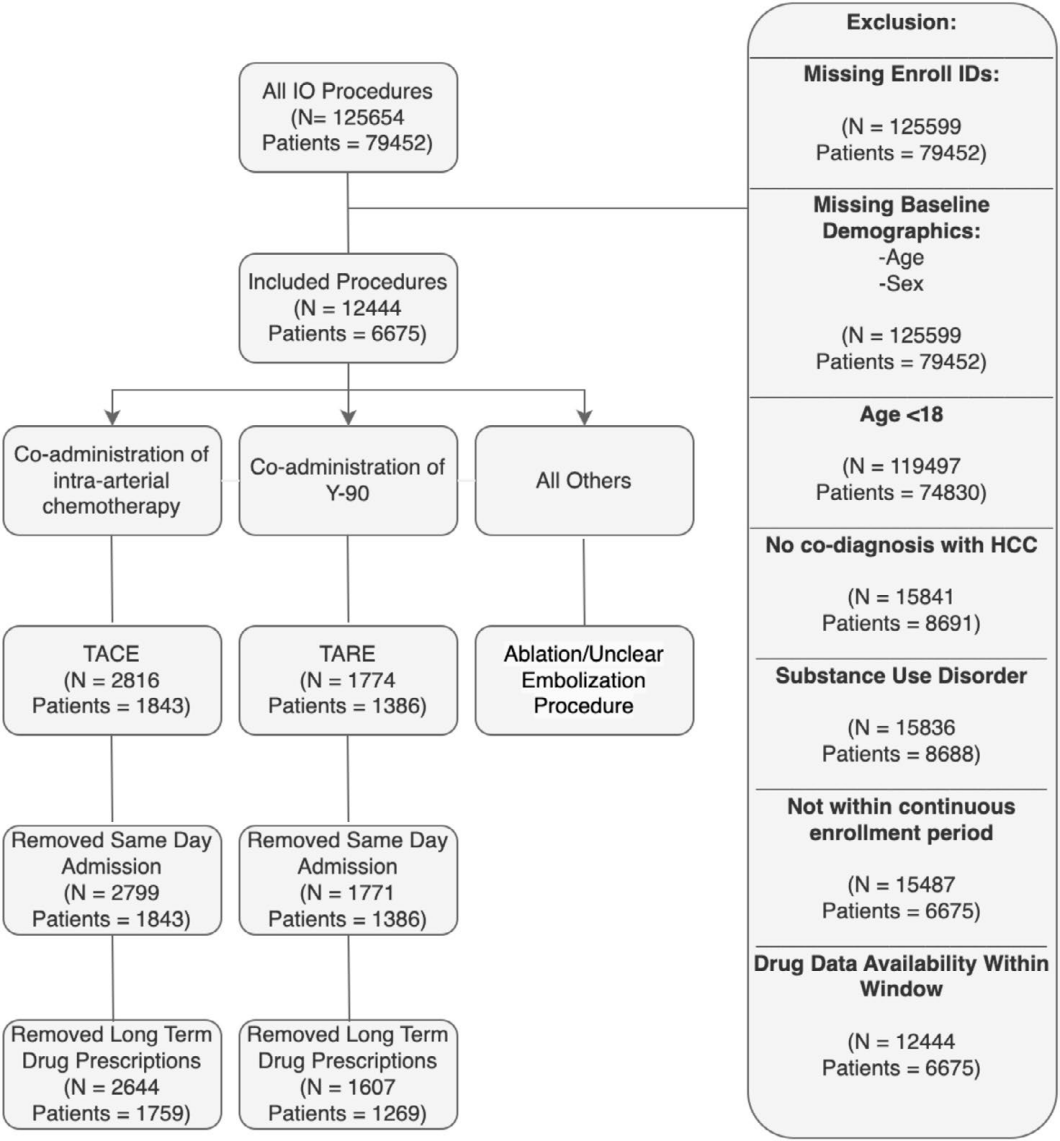
Flowchart of cohort construction. Interventional Oncology (IO) procedures identified by CPT and meeting the inclusion and exclusion criteria were stratified into the TACE and TARE cohorts based on HCPCS codes for co‐administration of intra‐arterial chemotherapy or yttrium‐90. Patients who were admitted on the day of the procedure, as well as those with prescriptions for long‐term pain medications, antiemetics, muscle relaxants, or steroids, were excluded to avoid confounding factors. TACE, trans‐arterial chemoembolization; TARE, trans‐arterial radioembolization; HCC, hepatocellular carcinoma; IO, interventional oncology.

The MarketScan database was queried using IO procedural codes (based on International Classification of Diseases Versions 9 and 10 [ICD‐9/10] and Common Procedural Terminology [CPT]). These codes along with the Healthcare Common Procedure Coding System (HCPCS) codes facilitated the stratification of patients into the TACE or TARE cohorts (Figure 1).

Patients were excluded if they were < 18 years old, had no concurrent HCC diagnosis, lacked accompanying drug data within 180 days before or after the procedure, had a diagnosis of substance use disorder, or had been prescribed an opioid, antiemetic, steroid, or non‐opioid pain drug within 7 days of the procedure for more than 7 days' supply indicating chronic use which may confound the analysis. A continuous enrollment period of 7 days before and after the procedure was mandated.

### 2.4 Variables

The outcome of interest was the presence of a hospital readmission or drug escalation event after 1 day but within 7 days of TACE or TARE. These thresholds were chosen because most instances of PES develop within 1–3 days post‐procedure [15]. Readmissions were identified in inpatient data, excluding those who were admitted for observation or full admission on the same day as the procedure. A drug escalation event was defined as a prescription of any medication on the day of the TARE or TACE procedure. The rates of hospital readmissions or drug escalation events were calculated as ratios of the number of patients with the respective outcome and the size of the respective cohort.

### 2.5 Statistical Analysis

Descriptive statistics for demographics included means and standard deviations for continuous variables and counts with percentages for categorical variables. Chi‐squared tests (and Fisher's Exact tests when necessary) and two‐sample *t*‐tests were used to compare the distributions of categorical and continuous variables, respectively, by treatment group.

Initially, bivariate logistic regression analyses were conducted to examine unadjusted associations between each discharge medication and the composite outcome. Variables demonstrating associations at *p* < 0.10 or deemed clinically relevant (age, sex, and Charlson Comorbidity Index [CCI]) were subsequently included in multivariable logistic regression models to adjust for potential confounding effects.

Multivariable logistic regression was then performed to evaluate the association between discharge medication prescriptions (opioids, non‐opioid medications, steroids, and antiemetics) and post‐procedural outcomes. To account for the non‐independence of repeated procedures within the same patient, robust standard errors clustered at the patient level were applied to address multiple observations per patient. Each medication class was entered as a binary variable indicating prescription at discharge, with age, sex, and CCI included as covariates. Adjusted odds ratios (aORs) and P‐values were estimated, and forest plots were generated to visually display effect estimates and 95% confidence intervals. Model adequacy was assessed using likelihood ratio tests, and multicollinearity was evaluated using variance inflation factors, all of which were < 2, indicating low inter‐variable correlation.

All analyses were carried out according to a pre‐specified plan. Continuous variables are summarized as *n*, mean (SD) as appropriate; binary variables as *n* (%). All tests were two‐sided with *α* = 0.05. Model assumptions were verified as described (goodness‐of‐fit via likelihood ratio tests; multicollinearity via VIFs). No adjustments for multiple comparisons were applied. Analyses were conducted from November 2023 to October 2025, using JMP Pro 17, SAS, version 9.4 (SAS Institute Inc., Cary, North Carolina), and JupyterLab, version 4.0.6 (Project Jupyter, New York, New York).

## 3 Results

### 3.1 Demographics and Patient Clinical Characteristics

The cohort included 3028 adult patients diagnosed with HCC who underwent a total of 4251 TACE or TARE procedures. Demographic and clinical characteristics of each cohort with respect to receiving discharge prescription medications of interest are summarized in Table 1. The observed outcomes of interest are demonstrated in Table 2.

**TABLE 1:** Demographic and clinical characteristics of patients undergoing TACE and TARE procedures.

	TACE		*p*	TARE		*p*
	With prescription discharge drugs	Without prescription discharge drugs		With prescription drugs	Without prescription drugs	
Cohort size; No.	378 procedures (299 distinct patients)	2266 procedures (1548 distinct patients)		645 procedures (557 distinct patients)	962 procedure (787 distinct patients)	
Age, years						
Mean (SD)	61.5 (8.1)	61.5 (9.1)	1.000	62.5 (10.4)	63.9 (10.7)	0.009
18–34; No. (%)	2 (0.53%)	9 (0.40%)	0.037	5 (0.78%)	6 (0.62%)	0.176
35–44; No. (%)	4 (1.06%)	56 (2.47%)		26 (4.03%)	32 (3.33%)	
45–55; No. (%)	48 (12.70%)	333 (14.70%)		87 (13.49%)	101 (10.50%)	
55–64; No. (%)	233 (61.64%)	1270 (56.05%)		323 (50.08%)	449 (46.67%)	
65+; No. (%)	91 (24.07%)	598 (26.39%)		204 (31.63%)	374 (38.88%)	
Sex						
Female; No. (%)	74 (19.58%)	519 (22.90%)	0.171	189 (29.30%)	230 (23.91%)	0.018
Male; No. (%)	304 (80.42%)	1747 (77.10%)		456 (70.70%)	732 (76.09%)	
Charlson Comorbidity Index (CCI); mean (SD)	4.1 (2.8)	3.9 (2.6)	0.195	4.3 (2.6)	4.5 (3.0)	0.156
Census region						
Northeast; No. (%)	40 (10.58%)	253 (11.17%)	0.948	101 (15.66%)	114 (11.85%)	< 0.001
North Central; No. (%)	90 (23.81%)	547 (24.14%)		144 (22.33%)	336 (34.93%)	
South; No. (%)	176 (46.56%)	1074 (47.40%)		272 (42.17%)	368 (38.25%)	
West; No. (%)	70 (18.52%)	381 (16.81%)		126 (19.53%)	126 (13.10%)	
Unknown; No. (%)	2 (0.53%)	11 (0.49%)		2 (0.31%)	18 (1.87%)	

*Note:* Demographics were calculated at the procedure level even though patients may have multiple TACE or TARE encounters. The same patient may be discharged with or without prescription drugs on separate encounters. *T*‐tests were used for continuous variables (age and Charlson Comorbidity Index), whereas Chi‐Squared Tests (and Fisher's Exact tests when necessary) were used on categorical variables. The term “distinct patients” refers to the number of individual patients involved in the cohort. Some patients underwent multiple procedures. Each patient was required to have an HCC co‐diagnosis to be included.

Abbreviations: TACE, trans‐arterial chemoembolization; TARE, trans‐arterial radioembolization.

**TABLE 2:** Comparison of patient outcomes following TACE and TARE procedures.

	TACE		*p*	TARE		*p*
	With prescription discharge drug (*n* = 378)	Without prescription discharge drugs (*n* = 2266)		With prescription drugs (*n* = 645)	Without prescription drugs (*n* = 962)	
Drug escalation						
Yes; No. (%)	55 (14.55%)	1316 (58.08%)	< 0.001	78 (12.09%)	279 (29.00%)	< 0.001
Readmission						
Yes; No. (%)	12 (3.17%)	53 (2.34%)	0.428	10 (1.55%)	17 (1.77%)	0.894
Drug escalation or readmission						
Yes; No. (%)	64 (16.93%)	1345 (59.36%)	< 0.001	88 (13.64%)	295 (30.67%)	< 0.001

Abbreviations: TACE, trans‐arterial chemoembolization; TARE, trans‐arterial radioembolization.

### 3.2 Patient Outcomes

#### 3.2.1 Drug Escalation

In the TACE cohort,14.6% of patients discharged with prescription drugs experienced a drug escalation as compared to 58.1% of those discharged without prescription drugs (Table 2). In the TARE cohort, drug escalation occurred in 12.1% of patients with prescription drugs at discharge, in contrast to 29.0% without.

#### 3.2.2 Readmission & Drug Escalation

Overall, readmission rates alone were similar between those with and without prescription drugs at discharge in both TACE (3.2% with vs. 2.3% without; *p* = 0.43) and TARE (1.6% with vs. 1.8% without; *p* = 0.89) cohorts. However, when examining the proportion of patients who have either a readmission or drug escalation, a higher proportion of patients experienced either event when discharged without prescription medication (TACE: 59.4%, *p* < 0.001; TARE: 30.7%, *p* < 0.001).

### 3.3 Top 5 Drug Prescription Combinations at Discharge

#### 3.3.1 TACE

Within the TACE cohort, the predominant drug class combination was an opioid and antiemetic, representing 48% of discharge prescriptions for patients following TACE. Subsequently, the opioid drug class alone was the second most common discharge drug class regimen, representing 28.3% of discharge prescriptions. The antiemetic drug class alone was the third most common discharge drug class regimen, representing 11.4% of discharge prescriptions (Table 3).

**TABLE 3:** Top 5 drug combinations by frequency for TACE and TARE.

#	TACE			TARE		
	Medication combination	Most frequent example	Frequency no. (%)	Medication combination	Most frequent example	Frequency no. (%)
1	Opioid and antiemetic	Ondansetron 4 MG × 9 pills Oxycodone 5 MG × 30 pills	181 (48%)	Opioid and Antiemetic	Acetaminophen/Oxycodone 325 MG‐5 MG × 50 pills Ondansetron 8 MG × 20 pills	157 (24.4%)
2	Opioid only	Oxycodone 5 MG × 30 pills	107 (28.3%)	Opioid, Antiemetic, and Steroid	Acetaminophen/Hydrocodone Bitartrate 325 MG‐5 MG × 30 pills Methylprednisolone 4 MG × 21 pills Ondansetron 4 MG × 30 pills	144 (22.4%)
3	Antiemetic only	Ondansetron 4 MG × 20 pills	43 (11.4%)	Opioid Only	Oxycodone 5 MG × 20 pills	85 (13.2%)
4	Opioid, steroid	Acetaminophen/Oxycodone 325 MG‐7.5 MG × 40 pills Prednisone 5 MG × 48 pills	13 (3.4%)	Opioid, Steroid	Methylprednisolone 4 MG × 21 pills Oxycodone 5 MG × 30 pills	80 (12.4%)
5	Opioid, antiemetic, steroid	Methylprednisolone 4 MG × 21 pills Ondansetron 4 MG × 20 pills Oxycodone 5 MG × 12 pills	13 (3.4%)	Antiemetic, Steroid	Methylprednisolone 4 MG × 21 pills Metoclopramide 5 MG × 120 pills	75 (11.6%)

Abbreviations: MG, milligrams; TACE, trans‐arterial chemoembolization; TARE, trans‐arterial radioembolization.

#### 3.3.2 TARE

Within the TARE patient cohort, the most common drug class combination at discharge was similar to that of TACE, with opioid and antiemetic drug classes, representing 24.4% of post‐TARE discharge prescriptions. The second most common drug class combination was comprised of the opioid, antiemetic, and steroid drug classes, representing 22.4% of discharge prescriptions. The third most common drug class prescription at discharge was an opioid alone, representing 13.2% of discharge prescriptions (Table 3).

### 3.4 Discharge Drug Prescription and Post‐Procedural Readmission or Drug Escalation

#### 3.4.1 TACE

Within the TACE cohort, prescription of discharge opioids and antiemetics was associated with lower odds of an escalation prescription or readmission after adjusting for age, sex, and CCI (Table 4). Patients prescribed opioids at discharge had an 83% lower odds of drug escalation or readmission (adjusted odds ratio [aOR] = 0.17, *p* < 0.001) than patients who were not prescribed opioids. Similarly, those prescribed antiemetics had 51% lower odds of the same outcomes (aOR = 0.49, *p* = 0.003) than patients who were not prescribed antiemetics. The prescription of steroids was not significantly associated with drug escalation or hospital readmission (aOR = 0.45, *p* = 0.10).

**TABLE 4:** Adjusted associations between discharge medications and drug escalation or admission in TACE and TARE patients.

Variable	aOR (95% CI)	*p*
TACE		
Discharge medication: opioid (reference: not prescribed opioid)	0.17 (0.12–0.24)	< 0.001
Discharge medication: non‐opioid pain drug (reference: not prescribed non‐opioid pain drug)	1.19 (0.16–8.73)	0.86
Discharge medication: steroid (reference: not prescribed steroid)	0.45 (0.17–1.18)	0.10
Discharge medication: antiemetic (reference: not prescribed antiemetic)	0.49 (0.31–0.78)	0.003
Sex (reference: male)	1.04 (0.85–1.27)	0.71
Age (years)	0.97 (0.95–0.98)	< 0.001
CCI	0.98 (0.94–1.03)	0.15
TARE		
Discharge medication: opioid (reference: not prescribed opioid)	0.41 (0.28–0.59)	< 0.001
Discharge medication: steroid (reference: not prescribed steroid)	0.63 (0.44–0.89)	0.007
Discharge medication: antiemetic (reference: not prescribed antiemetic)	0.65 (0.43–0.99)	0.04
Sex (reference: male)	1.00 (0.75–1.33)	0.99
Age (years)	0.98 (0.97–0.99)	< 0.001
CCI	1.01 (0.97–1.06)	0.77

*Note:* Discharge non‐opioid pain drug was removed from the analysis for TARE due to low counts resulting in the failure of the model to converge.

Abbreviations: aOR, adjusted odds ratio; CCI, Charlson Comorbidity Index; CI, confidence interval; TACE, trans‐arterial chemoembolization; TARE, trans‐arterial radioembolization.

#### 3.4.2 TARE

Within the TARE cohort, the prescription of opioids, antiemetics, and steroids at discharge was associated with lower odds of an escalation prescription or readmission after adjusting for age, sex, and the CCI (Table 4). Patients who were prescribed opioids upon discharge had 59% lower odds of the outcome (aOR = 0.41, *p* < 0.001) than those who were not prescribed opioids. Similarly, those prescribed antiemetics had 35% lower odds (aOR = 0.65, *p* = 0.04) than patients who were not prescribed antiemetics, and those prescribed steroids had 37% lower odds (aOR = 0.63, *p* = 0.007) of the same outcomes than patients who were not prescribed steroids.

**FIGURE 2:**
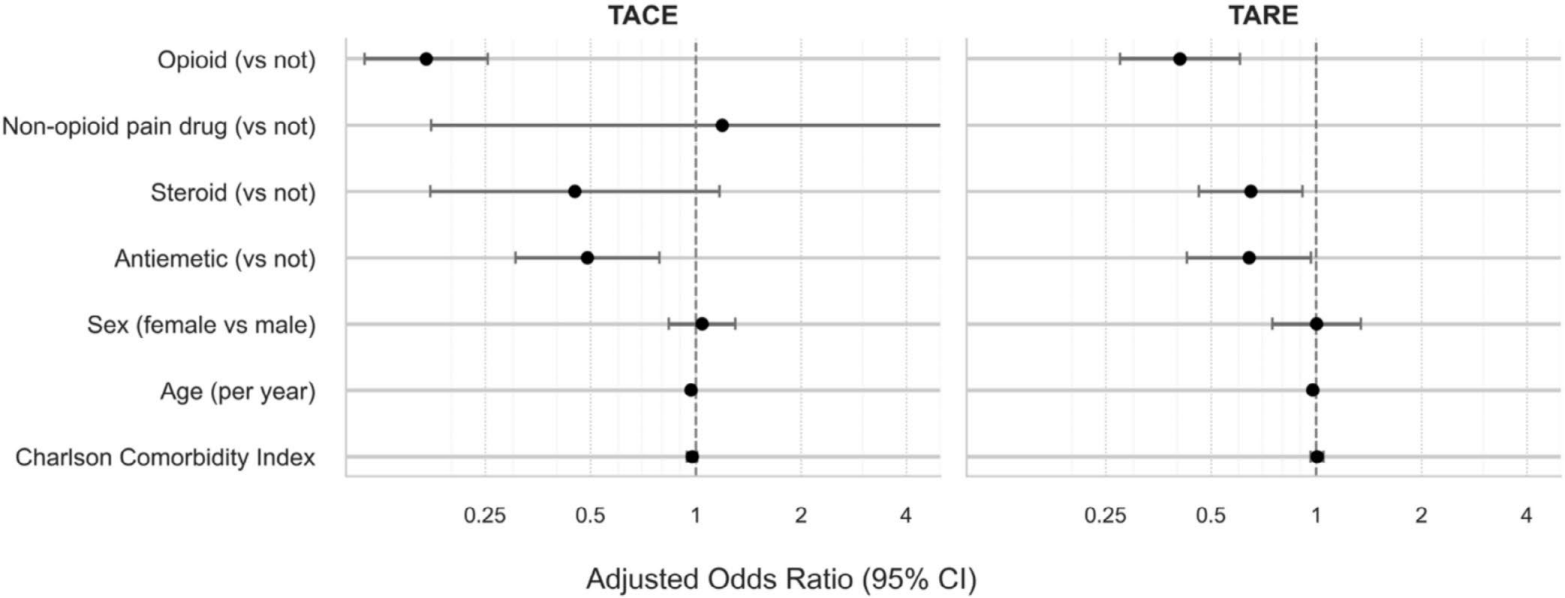
Association of discharge medications with 7‐day postprocedural escalation or readmission after TACE and TARE. Forest plots display aORs with 95% CIs from multivariable logistic regression using patient‐clustered robust standard errors and covariates (age, sex, CCI). The vertical dashed line denotes no effect (OR = 1). TACE, trans‐arterial chemoembolization; TARE, trans‐arterial radioembolization.

These associations are summarized visually in Figure 2, which displays adjusted odds ratios and 95% confidence intervals for each discharge medication class across the TACE and TARE cohorts. Model adequacy was evaluated using likelihood ratio tests comparing models with and without discharge medication variables, demonstrating significantly improved model fit with the inclusion of these variables (*p* < 0.001 for both TACE and TARE). Multicollinearity was assessed using variance inflation factors (VIFs), with all values < 2, indicating low inter‐variable correlation.

## 4 Discussion

This study explored the association between post‐TACE/TARE discharge medication regimens and escalation events, defined as (1) prescriptions targeting PES‐related symptoms or (2) admission to the inpatient setting following the post‐procedural window (24 h) and within a 7‐day period.

Patients who received prescription drugs at discharge were less likely to have a subsequent prescription drug escalation or a hospital readmission. While literature has attempted to characterize the various strategies for mitigating PES, it currently lacks evidence for optimal pain management practices in order to prevent PES. The findings thus provide novel insight into the post‐procedural symptomatology and the treatment strategies employed by the collective body of practitioners. Notably, drug prescription data at discharge—including drug categories, dosage, and quantities—offer implicit insights into prescriber anticipation regarding both the spectrum and potential severity of procedure‐related symptoms following a procedure. In this study's context, opioids are used to manage post‐embolization pain; antiemetics address nausea often associated with hepatic irritation or systemic inflammation; and steroids are prescribed to mitigate inflammatory responses. Importantly, post‐discharge new drug prescription claims serve as an indirect measure of patient symptomatology, as significant discomfort or adverse symptoms likely prompt patients to seek further consultation with their treating providers to obtain these prescriptions.

Demographic analysis of both TACE and TARE cohorts demonstrated a male majority and a majority of patients within the 55–64 age group, which is consistent with the literature that reports a U.S. median age of 60–64 for a diagnosis of HCC in a predominant 10.4 of 100,000 male persons (versus 2.9 per 100,000 females) [16]. The slight inverse relationship between age and the likelihood of drug escalation or admission may appear counterintuitive. However, examining the patient cohort experiencing either a drug escalation or hospital readmission shows a lower average age but more comorbidities, whereas those without the outcome exhibited a higher age with fewer comorbidities. This pattern may reflect a “healthy worker survivor effect” [17], wherein the older individuals selected for TACE or TARE procedures represent a subgroup that is comparatively healthier and thus capable of retaining their employment‐based health insurance and of undergoing these interventions despite advanced age.

Based on commonly prescribed medications at discharge, clinical practices may both share and differ in their approach to prophylactic preparedness for PES‐related symptoms, depending on whether TACE or TARE is used. For TACE, the most frequently prescribed discharge medication was a combination of the opioid and anti‐emetic drug classes, specifically Oxycodone 5 mg and Ondansetron 4 mg. TARE patients were most commonly prescribed both an opioid and an anti‐emetic at discharge, which is Acetaminophen/Oxycodone 325–5 mg and Ondansetron 8 mg. The higher prescription dose of ondansetron in the TARE group may suggest a greater expectation of PES‐related nausea following TARE as a result of its radiation‐based mechanism. Additionally, the inclusion of steroids in commonly prescribed discharge medications is not apparent until the fourth most common drug for TACE, whereas it is immediately apparent in the second most common drug for TARE. For TARE, clinicians most frequently prescribe methylprednisolone, which may indicate a prioritization and concern for mitigating longer‐acting inflammatory sequelae of radiation exposure to both malignant and benign tissues. The consistent use of anti‐inflammatory medication across both patient groups suggests a noncodified recognition and treatment of inflammation as a central driver of PES.

In the post‐operative period, patients who experience severe pain are impeded physically and mentally. Inadequate pain management results in worse outcomes and causes patients to request more medications to alleviate their symptoms, leading to more frequent visits [18, 19]. Unfortunately, policies aimed at reducing opioid misuse, such as the enforcement of Prescription Drug Monitoring Programs, have had unintended repercussions contributing to increased discrimination and a lack of appropriate pain management [20]. Drug escalations reflect patients seeking solutions for their symptoms and have been shown to be a key indicator of worsening pain, which is predictive of pain status, disability, and overall outcomes [21]. Therefore, post‐operative pain drug escalations show value as being a marker of pain status as well as an indicator of the patient's recovery trajectory.

### 4.1 Opioids

This analysis presents a compelling case for reevaluating pain management strategies post‐TACE and TARE procedures. We observed 83% and 59% lower odds of prescription escalation or readmission when opioids were prescribed upon discharge for TACE and TARE, respectively. These findings are consistent with Zhou et al.'s randomized clinical trial [22], which underscored the efficacy of oral opioids in superior pain management post‐TACE. Prajapati et al.'s observations support this finding as well, with effective mitigation of PES‐related abdominal pain with oral opioids [23]. Interestingly, while TARE‐associated pain is reportedly milder and treatable with over‐the‐counter medications [24], our data suggested a non‐negligible benefit from post‐discharge opioid prescriptions, challenging the current conservative approach observed in European centers, where only a minority of centers employ opioid prescription at discharge [5]. Together, these findings advocate for a protocolized approach to pain management post‐TACE or TARE with the integration of oral opioids as part of the discharge plan to preemptively address pain and potentially reduce the rate of post‐procedural escalation prescriptions or admissions.

### 4.2 Anti‐Emetics

This study found 55% and 35% lower odds of escalation/readmission with an anti‐emetic prescription for TACE and TARE, respectively, at the time of discharge. The use of discharge anti‐emetics in TACE has been reported favorably in several small‐sample studies [25, 26]. Interestingly, European survey data reveal that only approximately one‐third of cancer centers prescribe discharge anti‐emetics [27].

### 4.3 Steroids

In this analysis, the observed 56% and 37% lower odds of having an escalation event or readmission with steroid prescriptions at discharge for TACE and TARE, respectively, support the prescription of steroids at discharge. However, the conclusions of previous literature are split. Steroids have been found to be useful in reducing symptoms related to PES [28, 29, 30], but others did not find any significant effect on symptoms or outcomes post‐TACE [31, 32].

## 5 Limitations

Limitations of this study stem from the constraints of the data used, as they only encompass beneficiaries of employer‐sponsored health insurance. The data do not include individuals with other types of insurance, those who pay for prescriptions fully out‐of‐pocket, or the uninsured. These differences should be considered when extrapolating our findings to broader populations. While there is an inherent possibility of selection bias with the use of data on beneficiaries of employer‐sponsored insurance, we applied careful inclusion and exclusion criteria to ensure that the study cohort represented the target population and mitigated the influence of post‐exposure interventions to isolate the association between discharge medications and outcomes of interest. Exposure was simplified to the presence of discharge medication classes, allowing for clear, consistent definitions and straightforward comparisons between medication types.

Another limitation of the study is the use of all‐cause readmissions. Due to limitations in medical coding, we were unable to isolate readmissions specifically related to PES, and thus had to rely on all‐cause readmissions.

The absence of specific clinical data, including the intraoperative use of intravenous opioids, antiemetics, and steroids, HCC staging, etiology, presence of extrahepatic metastasis and their sites, vascular invasion, performance status, concurrent treatments, or number of locoregional therapies, presents a limitation in fully understanding their role in PES‐related outcomes. Previously described predictors of PES, such as large tumor burden, use of drug‐eluting embolics, and doxorubicin dose [25, 33], were not included in the data. However, this analysis contributes to the existing knowledge base by highlighting prescription patterns associated with PES‐related outcomes. Finally, due to the observational nature of this study, residual confounding remains a possibility.

## 6 Conclusion

This claims‐based cohort study provides insight into physician decision‐making processes and prioritization of patient symptomatology. The evaluation of physicians' tendency towards prescribing opioids, anti‐emetics, and steroids provides critical and implicit insights into clinical strategies and anticipatory measures to address treatment‐related pain. While we have identified the most common drug combinations prescribed, further work is needed to determine optimal opioid type, duration of prescription, and dosage, as no current consensus exists. This may also require additional research into the characterization of PES. Finally, future work may examine the effect of opioid‐related legislation and its impact on provider prescription patterns, and ultimately, on patient care.

## Author Contributions

Conceptualization: Hanzhou Li, John T. Moon, Deepak Iyer. Methodology: Hanzhou Li, John T. Moon, Michal Horný. Formal analysis: Hanzhou Li, John T. Moon, Menelaos Konstantinidis, Michal Horný. Data curation: Hanzhou Li, John T. Moon, Nicholas Lima. Investigation: Hanzhou Li, John T. Moon, Nicholas Lima, Michal Horný. Writing – original draft: Hanzhou Li, John T. Moon, Nathan Sim, Nicholas Lima. Writing – review and editing: Hanzhou Li, John T. Moon, Michael Prologo, Judy Gichoya, Janice Newsome, Zachary Bercu, Sonia Benenati, Michal Horný. Visualization: Hanzhou Li. Funding acquisition: Zachary Bercu, Hanzhou Li, John T. Moon, Janice Newsome, Deepak Iyer. Resources: Hanzhou Li, John T. Moon, Nathan Sim. Supervision: Janice Newsome, Zachary Bercu. Project administration: Hanzhou Li, John T. Moon. Validation: Hanzhou Li, John T. Moon.

## Funding

This study was supported in part by the American College of Radiology Harvey L. Neiman Health Policy Institute (HPI) under award number UL1TR002378. Funding was not involved in the design and conduct of the study or any part of the writing, review, or submission processes.

## Ethics Statement

This secondary analysis of de‐identified claims data from the Merative MarketScan Databases was reviewed and approved by the Institutional Review Board.

## Consent

Patient consent was waived because all data were de‐identified.

## Conflicts of Interest

The authors declare no conflicts of interest related to this work. The following include non‐relevant financial disclosures for the authors: Janice Newsome is an educational consultant for Cook Medical and is on the scientific advisory board for Boston Scientific, CareStream, and Road2IR. Zachary Bercu is a speaker and educational consultant for Terumo Medical Corporation and vice president and board member of the Southeastern Angiographic Society. Michal Horný reported receiving research funding from Arnold Ventures, Health Care Cost Institute, the Commonwealth Fund, the Centers for Disease Control and Prevention, and the National Center for Advancing Translational Sciences for unrelated work; receiving travel support and speaking honoraria from Georgetown University, Charles University, Masaryk University, and the Czech Academy of Sciences; receiving payments from the American Medical Association for statistical review of manuscripts; and receiving consulting fees from VBID Health LLC.

## Data Availability

The data underlying this article are available from the Merative MarketScan Research Databases under a data‐use license and are not publicly available. Information on data access is available at https://www.merative.com/products/marketscan (Research Databases program).
